# Localization of *Salmonella* and albumin-IL-2 to the tumor microenvironment augments anticancer T cell immunity

**DOI:** 10.1186/s12929-022-00841-y

**Published:** 2022-08-12

**Authors:** Yu-Jui Kung, Brandon Lam, Ssu-Hsueh Tseng, Alana MacDonald, Hsin-Fang Tu, Suyang Wang, John Lin, Ya Chea Tsai, T. C. Wu, Chien-Fu Hung

**Affiliations:** 1 Department of Pathology, Johns Hopkins School of Medicine, Baltimore, MD USA; 2 Graduate Program in Immunology, Johns Hopkins School of Medicine, Baltimore, MD USA; 3 Stanford University School of Medicine, Stanford, CA USA; 4 Department of Oncology, Johns Hopkins School of Medicine, Baltimore, MD USA

**Keywords:** Colon cancer, Albumin, Interleukin-2 (IL-2), Salmonella

## Abstract

**Background:**

For centuries, microbial-based agents have been investigated as a therapeutic modality for the treatment of cancer. In theory, these methods would be cheap to produce, broadly applicable in a wide array of cancer types, and could synergize with other cancer treatment strategies. We aimed to assess the efficacy of combining microbial-based therapy using *Salmonella* SL7207 with interleukin-2 (IL-2), a potent immunostimulatory agent, in the treatment of murine colon carcinoma.

**Methods:**

Female BALB/c mice were implanted subcutaneously with CT26 tumors, a model of colon carcinoma. Mice bearing tumors were selected and administered Albumin-IL-2 (Alb-IL2), a fusion protein, for further analysis of anticancer effect.

**Results:**

We demonstrated that *Salmonella* SL7207, a genetically modified strain of *Salmonella enterica* serovar Typhimurium, preferentially accumulates in the tumor microenvironment, potentiating it to stimulate localized innate immunity. We delivered IL-2 as a fusion protein, Alb-IL2, which we demonstrate to have preferential accumulation properties, bringing it to the tumor and secondary lymphoid organs. Treatment of tumor-bearing mice with *Salmonella* + Alb-IL2 leads to superior tumor control and enhanced overall survival compared to controls. When assessing immunological factors contributing to our observed tumor control, significantly enhanced T cell population with superior effector function was observed in mice treated with *Salmonella* + Alb-IL2. We confirmed that these T cells were indispensable to the observed tumor control through antibody-mediated T cell depletion experiments.

**Conclusions:**

These findings highlight the ability of *Salmonella* + Alb-IL2 to serve as a novel therapeutic approach to induce T cell-mediated antitumor immunity and exert long-term tumor control in a murine model of cancer.

**Supplementary Information:**

The online version contains supplementary material available at 10.1186/s12929-022-00841-y.

## Background

Beginning in the nineteenth century, clinicians began to correlate evidence of pathogenic infection with regression of tumors in cancer patients [1]. These initial observations were the basis for numerous basic and translational studies investigating the use of infectious agents for the treatment of cancer [2]. However, two main factors curtailed the development of infectious agents as a cancer therapeutic: off-target toxicity associated with administering infectious agents to patients and the rapid development of other agents, such as chemotherapy, with unprecedented clinical success compared to other available strategies at the time [1].

Recently, there has been a resurgence in interest to utilize infectious agents for the treatment of cancer with hope that modern technological advancements could shed light on the mechanism of action, overcome the previous toxicity issues, and ultimately lead to a successful therapeutic strategy. Strategies range from systemic infection with a plethora of microbial species to engineered organisms programmed to deliver chemoattractants, chemotherapeutics, or other cytotoxic agents to specific tissues [3].

*Salmonella* species have been explored extensively for their ability to promote tumor control in numerous contexts and remain a promising microbial-based treatment modality [4]. *Salmonella* SL7207 is a genetically modified strain of *Salmonella enterica* serovar Typhimurium that exhibits preferential accumulation in the tumor microenvironment (TME) [5]. This provides the opportunity to deliver *Salmonella* directly to the TME, which limits some off-target systemic cytotoxicity while triggering localized innate immune sensing pathway.

A potential approach to further reduce toxicity and optimize efficacy of *Salmonella* SL7207 treatment in cancer would be through combination therapy with other treatment modalities. This would allow for less *Salmonella* to reach clinically relevant therapeutic levels, as the response would dovetail on the effect of the other modality. An attractive combinatorial approach would be one that incorporates a T cell-stimulating agent. This is because mechanistically, cancer treatment with *Salmonella* depends on the induction of T cell immunity for long-term control [4].

IL-2 is a cytokine known for its indispensable role in T cell activation and expansion [6]. IL-2 is regarded as the first effective immunotherapy for the management of human cancer [7]. Upon engagement of the T cell receptor with antigen in the context of major histocompatibility complex (MHC) and costimulatory molecules, IL-2 is produced, which subsequently drives T cell proliferation through autocrine signaling or by binding and presentation on adjacent CD25 + antigen presenting cells [6]. For these reasons, IL-2 has been widely implemented as a strategy to enhance anti-tumor T cell responses. In clinical studies, objective responses were observed in approximately 15% of patients treated with IL-2, with a subset of patients achieving durable, long-term control [8, 9]. As a result, IL-2 received FDA approval in 1992 and 1998 for metastatic renal cell carcinoma and melanoma, respectively [7].

One challenge of using IL-2 therapeutically is its short serum half-life of about ~ 7 min in humans [10]. To address this, Albumin-IL-2 (Alb-IL2) fusion proteins can be used. Albumin is an abundant, ubiquitous protein characterized by a long half-life of approximately 3 weeks due to a pH dependent, neonatal Fc receptor (FcRn) mediated recycling mechanism [11]. In addition, albumin can readily infiltrate tumor tissue and has been shown to enhance intra-tumoral accumulation [12]. These properties have led to investigation of albumin as a vehicle for tumoral drug delivery [13]. When specifically considering Alb-IL2, significantly improved pharmacokinetic properties and increased uptake in lymph nodes, spleen, and liver of mice was observed [14, 15]. Melder et al. compared the bioactivity between Albuleukin and recombinant IL-2 and concluded that both competed for IL-2 receptors that induced the production of interferon γ (IFNγ) from mouse and human T cells [15]. They investigated the therapeutic potential of Alb-IL2 and demonstrated improved survival outcomes, reduced tumor growth, and significant infiltration of T cells in tumors when compared to IL-2. The stronger anti-tumor effects observed for Alb-IL2 compared to IL-2 could be attributed to its enhanced half-life which extends contact with IL-2R + lymphocytes, such as T cells, and ultimately potentiates T cell immunity [16].

In this study, we investigated the use of bacteria therapy, namely, SL7207, in combination with Alb-IL2 immunotherapy as a novel approach for the treatment of cancer. We hypothesize that localization of *Salmonella* to the TME will generate a local source of inflammation that will drive effective immune activation and priming of T cells while administration of Alb-IL2 will promote enhanced activation and proliferation of tumor reactive T cell clones. Together, these factors would synergize and lead to durable responses and tumor control.

## Materials and methods

### Ethics approval

The housing and handling of mice follow guidelines established by Johns Hopkins Medical Institutions Animal Care and Use Committee and the National Institutes of Health. Animals are monitored daily for infection and other illnesses by trained animal technicians. Only trained laboratory personnel and animal technicians were allowed to handle laboratory animals. All individuals handling mice were registered to protocols at the Johns Hopkins Animal Care and Use Committee.

### Mice

Six-week old female BALB/c mice were purchased from Taconic Biosciences (Cambridge City, IN, USA). All mice were housed in specific-pathogen free conditions at the Johns Hopkins University School of Medicine Oncology Animal Facility in Koch Cancer Research Building II (Baltimore, MD). All animal procedures followed approved protocols from the Johns Hopkins Institutional Animal Care and Use Committee and were in accordance with recommendations for proper use and care of laboratory animals.

### Cell culture

CT26 tumor cells were grown in vitro in RPMI-1640 media supplemented with 10% fetal bovine serum, 50 units/mL of penicillin/streptomycin, 2 mM of L-glutamine, 1 mM of sodium pyruvate, and 2 nM of non-essential amino acids, and grown at 37 °C, 5% CO_2_. Cells were passaged using 0.05% Trypsin/EDTA after reaching 90 + % confluency. Cells were enumerated using a Countess II from Invitrogen (Carlsbad, CA, USA) and washed extensively with PBS before use. *Salmonella* SL7207 was kindly provided by Dr. Bruce Stocker (Stanford University, Stanford, CA) and grown under the recommended conditions in LB broth at 37 °C. Cells were harvested for injection when O.D. 600 reading was ~ 0.5 and washed extensively with PBS prior to injection.

### Generation of luminescent *Salmonella* SL7207

*Salmonella* SL7207 was electroporated with 100 ng of pGEN-luxCDABE. pGEN-luxCDABE was a gift from Harry Mobley (Addgene plasmid # 44918; http://n2t.net/addgene: 44918; RRID:Addgene_44918). A single ampicillin resistant clone was selected, grown, and used where indicated.

### Generation of Alb-IL2 protein constructs

For the generation of pcDNA3- Alb-IL2, mouse IL2 was first amplified via PCR with pcDNA3-IL2 described previously [17] and the following primers: 5′AAAgaattcGCACCCACTTCAAGCTCC-3′ and 5′- AAACTTAAGTTATTGAGGGCTTGTTGA -3′. The amplified product was then cloned into the EcoRI/Afl II sites of pcDNA3-Alb [18]**.**

The plasmid constructs were confirmed by DNA sequencing. Alb-IL2 proteins were expressed using Expi293F expression system kit from Thermo Fisher Scientific (Waltham, MA, USA) according to manufacturer’s instructions. Expi293F cells were transfected with Alb-IL2. Proteins were purified by HiTrap Albumin column from GE Healthcare Life Sciences (Marlborough, MA, USA).

### Tumor challenge and treatment

For CT26 cell challenge, 2 × 10^5^ tumor cells were injected subcutaneously into six-week-old female BALB/c mice. 5 × 10^6^ *Salmonella* were injected intravenously at the indicated time points. Mice were injected intravenously with 50 µg of Alb-IL2 protein prepared in sterile PBS. Tumors were measured (1/2 ×  (long diameter  × short diameter^2^)) at the indicated points and were euthanized in accordance with the approved animal care and use protocol.

### Preparation of single cell suspensions for flow cytometric analysis

For tumor specific immune profiling, tumor draining lymph nodes (LN) were collected from CT26 tumor bearing mice that were treated as indicated. The LNs were dissociated using a syringe plunger and 70uM filter and washed using fluorescence-activated cell sorting (FACS) buffer from Thermo Fisher Scientific. Cell suspensions were then RBC lysed, washed, and resuspended in FACS buffer for downstream analysis.

### Flow cytometric acquisition and analysis

For all experiments utilizing flow cytometry, single cell suspensions were prepared and extensively filtered prior to acquisition. Single staining controls of ultracomp beads from Thermo Fisher Scientific were used to set compensation matrix for each experiment. Negative gates were determined using appropriate Fluorescence Minus One or isotype controls as appropriate. Prior to antibody staining, Zombie Aqua live/dead from BioLegend (San Diego, CA, USA) was used to dead cells. Fc Block was used prior to antibody staining. Antibody and tetramer dilutions were determined through titration. All samples were acquired on a 13-color B-Y-R-V CytoFLEX S from Beckman Coulter (Brea, CA, USA). Compensation was generated using single-staining controls and CytExpert 2.0 software from Beckman Coulter. Analysis was performed using either CytExpert 2.0 or FlowJo v10 from BD Biosciences (Franklin Lakes, NJ, USA). All antibodies used in the analysis can be found in Table 1.

**Table 1:** Antibodies

Antibody	Catalog #	Manufacturer
InVivoMAb anti-mouse CD4	BE0003-1	Bio X Cell
InVivoMAb anti-mouse CD8a	BE0117	Bio X Cell
OVA Tetramer-PE	TB-5001-M	MBL
CD8a-FITC	100706	Biolegend
IFNγ-APC	505809	Biolegend
TNFα-BV650	506333	Biolegend
CD4-BV785	100453	Biolegend
IL2-PE	503807	Biolegend
CD3mc-PE-Cy7	100219	Biolegend
CD45-Alexa Fluor 700	147715	Biolegend

### Intracellular staining

To stain for intracellular cytokines, samples were incubated with PMA/Ionomycin cell stimulation cocktail and Brefeldin A + Monensin Golgi Plug from Thermo Fisher Scientific for 4 h at 37 °C. Cells were then collected and prepared as described in the flow cytometry section. Prior to intracellular staining, cells were permeabilized using eBioscience Foxp3 / Transcription Factor Staining Buffer Set from Thermo Fisher Scientific and stained for intracellular cytokines.

### Generation of fluorescent Alb-IL2

Alb-IL2 was labeled using Alexa Fluor 647 NHS Ester (Succinimidyl Ester) from Thermo Fisher Scientific according to manufacturer’s instructions. Free dye was removed using a 7000 Da MW Zebra Spin Desalting Column from Thermo Fisher Scientific.

### In vivo imaging system (IVIS) imaging

The trafficking of Alexa-647-Alb-IL2 and luminescent *Salmonella* SL7207 was observed using IVIS Series 2000 from PerkinElmer (Waltham, MA, USA). Alb-IL2 was labeled using Alexa Fluor 647 NHS Ester (Succinimidyl Ester) from Thermo Fisher Scientific according to manufacturer’s instructions. 50ug of protein was injected intravenously through the retroorbital sinus. After 18 h, mice were euthanized and the tumor, spleen, lymph nodes, and liver were removed and immediately imaged via the IVIS Spectrum with the following settings: 10 s exposure, excitation filter blocked, emission filter open, FOV 22.7, Binning: 8. Fluorescent signals from Alexa-647-Alb-IL2 were quantified as average radiance using Living Image 3.0 Software by Xenogen (Alameda, CA, USA). Bioluminescence imaging of Luminescent *Salmonella* SL7207 was conducted on the IVIS Spectrum with the following settings: 5 s exposure, excitation filter blocked, emission filter open, FOV 22.7, Binning: 8. Luminescent signals were quantified as average radiance using Living Image 3.0 Software by Xenogen.

### Statistical analysis

All data are expressed as means ± standard error of the mean (S.E.M). Results for flow cytometry analysis, IVIS imaging, and tumor treatment experiments were evaluated by analysis of variance (one-way ANOVA) and the Tukey–Kramer multiple comparison test where appropriate. Comparisons between individual data points were made using Student’s t-tests. Survival analysis was performed using Kaplan–Meier survival curves and log-rank tests. All P values < 0.05 were considered significant. Of note, *, **, ***, and *** indicate P values less than 0.05, 0.01, 0.001, and 0.0001 respectively. NS, not significant. All statistical calculations were performed in GraphPad Prism 9.

## Results

### *Salmonella* + Alb-IL2 demonstrated superior tumor control and extended survival compared to monotherapy controls

**Fig. 1:**
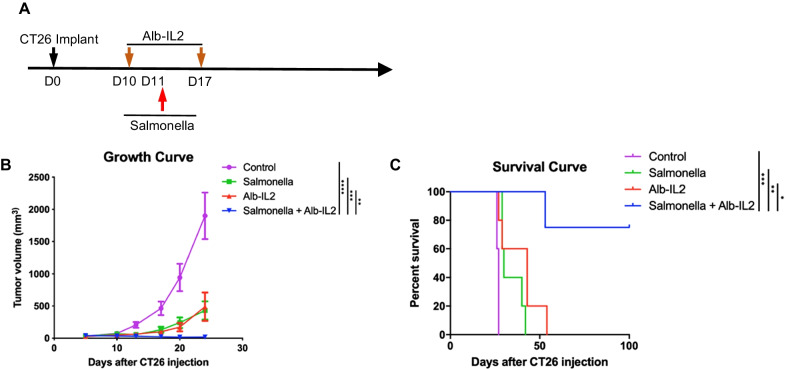
*Salmonella* + Alb-IL2 leads to superior tumor control and extended survival. **a** Schematic of experimental design. Briefly, BALB/c mice were inoculated with 5 × 10^5^ CT26 tumor cells subcutaneously. 10 days later, following establishment of tumors, mice were administered 50 µg of Alb-IL2. 1 day later, mice were treated with 5 × 10^6^ *Salmonella* intravenously. Mice were treated again with 50 µg of Alb-IL2 on day 17. **b** Tumor growth and **c** survival curve following the described treatment protocol. N = 5 mice per group. Data is represented by mean ± SEM. P values were calculated by ordinary one-way ANOVA with the Tukey–Kramer multiple comparison test, and P < 0.05 is considered statistically significant. *,**,***, and **** indicate p values less than 0.05, 0.01, 0.001, and 0.0001, respectively

Previous studies have reported that Alb-IL2 demonstrated modest anti-tumor effects [14–16] driven by T cell expansion and effector function. In the current study, we sought to evaluate the therapeutic efficacy of *Salmonella* and Alb-IL2 alone or in combination for the treatment of cancer. Female BALB/c mice were implanted subcutaneously with CT26 tumors, a model of colon carcinoma. Mice bearing equal tumors were selected and administered Alb-IL2 on days 10 and 17, and *Salmonella* SL7207 on day 11 intravenously (Fig. 1a). We reasoned that intratumoral colonization of *Salmonella* would broadly trigger innate immune sensing pathways and act as an in-situ adjuvant for tumor-specific T cell clones in the TME. Subsequent administration of Alb-IL2 will support the expansion and effector function of tumor-specific T cells. Treatment with *Salmonella* + Alb-IL2 resulted in significantly reduced tumor growth compared to *Salmonella* or Alb-IL2 treatment alone (Fig. 1b, Additional file 1: Fig. S1). This significant antitumor effect translated into improved survival of tumor-bearing mice, with 75% of mice treated with *Salmonella* and Alb-IL2 surviving past 100 days (Fig. 1c). Mice treated with *Salmonella* or Alb-IL2 alone showed reduced tumor growth. However, these mice succumbed to tumor burden by day 50. We also proved Salmonella + Alb-IL2’s ability to exert tumor control in the TC-1 tumors, a murine model of HPV-associated cancer (Additional file 1: Fig. S2). Together, this data suggests that *Salmonella* + Alb-IL2 is an effective approach to reduce tumor burden compared to single agent treatments.

### *Salmonella* SL7207 preferentially accumulates in murine tumors

**Fig. 2:**
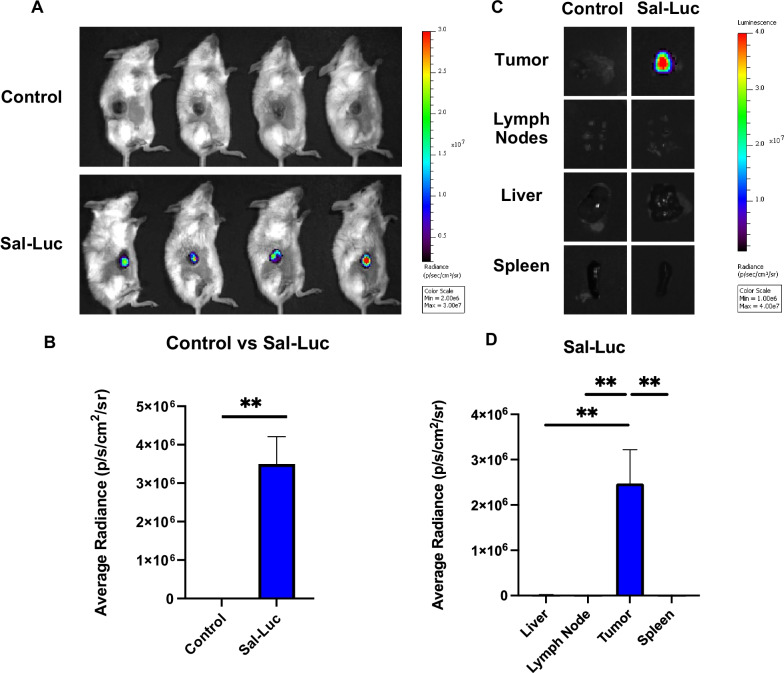
*Salmonella* SL7207 exhibits preferential colonization in the TME. **a** Mice were injected 5 × 10^6^ *Salmonella* SL7207 intravenously through the retroorbital sinus. After 18 h, mice were imaged via IVIS imaging. **b** Quantification of luminescent signal in the tumor. N = 4 mice per group. Data is represented by mean ± SEM. The p value was calculated by unpaired t-test and P < 0.05 is considered statistically significant. **Indicates p values less than 0.01. **c** Mice were injected 5 × 10^6^ *Salmonella* SL7207 intravenously through the retroorbital sinus. After 18 h, the mice were euthanized and tumors, lymph nodes, livers, and spleen were removed and imaged via IVIS imaging. **d** Quantification of the luminescent signals in the above organs. N = 4 mice per group. Data is represented by mean ± SEM. P values were calculated by ordinary one-way ANOVA with the Tukey–Kramer multiple comparison test, and P < 0.05 is considered statistically significant. * and ** indicate p values less than 0.05 and 0.01. Sal-Luc is short for luminescent *Salmonella* SL7207

Having observed the antitumor effects of *Salmonella* SL7207 administration, we wanted to gauge its ability to accumulate in our tumor system. CT26 tumor-bearing BALB/c mice were injected intravenously with luminescent *Salmonella* SL7207. Mice were imaged 18 h post-injection using the IVIS. When analyzing luminescent activity following whole body imaging, *Salmonella* treated mice (bottom) displayed robust luminescent activity in the anatomical region of the implanted CT26 tumor (Fig. 2a). To confirm the source of this signal, the mice were euthanized and tissues were collected for ex vivo imaging. Significantly more luminescent signal was observed in the tumor tissue, while minimal signal was detected in the lymph nodes, liver, and spleen (Fig. 2c, d). This confirms the preferential accumulation of *Salmonella* to the tumor, affirming our ability to deliver *Salmonella* directly to the TME and potentially support anti-tumor responses.

### Alb-IL2 traffics to the tumor and secondary lymphoid organs

**Fig. 3:**
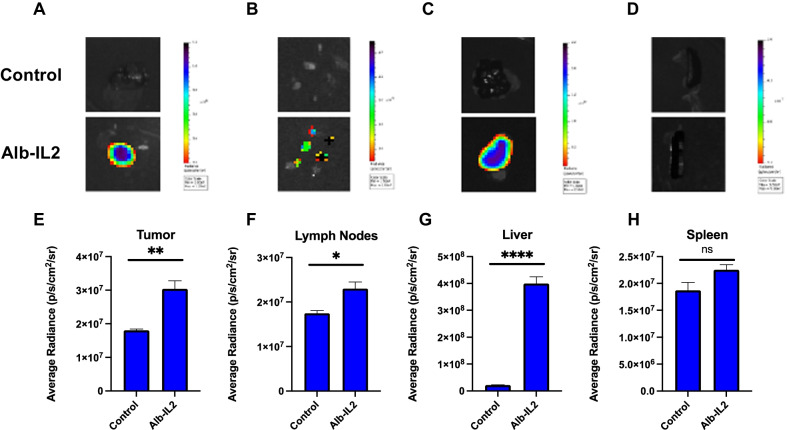
Accumulation of Alb-IL2 in the tumor and secondary lymphoid organs. BALB/c mice were inoculated with 5 × 10^5^ CT26 tumor cells subcutaneously. On day 15, mice were injected intravenously through the retroorbital sinus with Alexa-647 labeled Alb-IL2. After 18 h, the mice were euthanized and (**a**) tumor, (**b**) lymph nodes, (**c**) liver, and (**d**) spleen were collected and imaged via IVIS imaging. Quantification of the fluorescence signal of (**e**) tumor, (**f**) lymph nodes, (**g**) liver, and (**h**) spleen**.** N = 4 mice per group. Data is represented by mean ± SEM. P values were calculated by ordinary one-way ANOVA with the Tukey–Kramer multiple comparison test, and P < 0.05 is considered statistically significant. *,**,***, and **** indicate p values less than 0.05, 0.01, 0.001, and 0.0001, respectively. NS, not significant

First, we analyzed for the pSTAT5 levels of rested memory CD8 + T cells stimulated with Alb-IL2 to confirm its bioactivity (Additional file 1: Fig. S3). Next, given that albumin fusion is known to impact in vivo tissue distribution, we wanted to characterize the trafficking pattern of the Alb-IL2 fusion protein. Alb-IL2 was labeled using a succinimidyl ester labeling strategy to tag Alexa-647. CT26 tumor-bearing BALB/c mice were injected intravenously with PBS as control or 50 ug of Alexa-647-Alb-IL2. Mice were euthanized 18 h post injection and the tissues were used for fluorescent imaging studies by IVIS. When analyzing the fluorescence activity, Alb-IL2 demonstrated preferential trafficking to the tumor tissue (Fig. 3a,b). Significant fluorescent signal was also observed in the lymph nodes, livers, and spleens of mice treated with Alb-IL2 (Fig. 3c–h). There was an absence of fluorescent activity in the kidneys and lungs 18 h post injection, which is consistent with previous biodistribution and kinetics studies on Alb-IL2 [14]. Given this, it is possible that accumulation of Alb-IL2 in the tumor and lymph node could be playing a role in the observed antitumor responses.

### *Salmonella* + Alb-IL2 treatment enhances T cells immunity in the TME

**Fig. 4:**
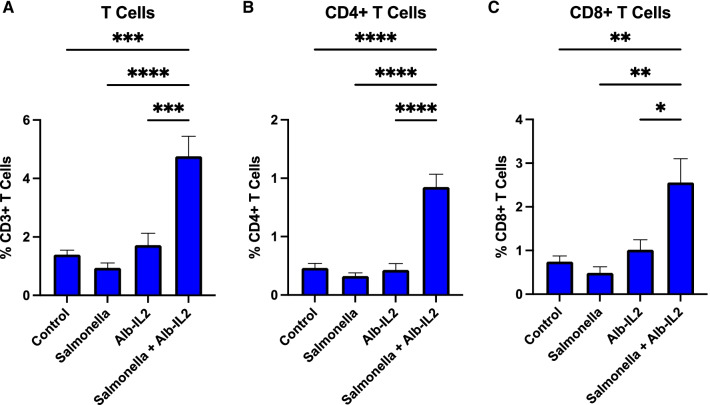
*Salmonella* + Alb-IL2 treatment increases the frequency of T cells in the TME. BALB/c mice were inoculated with 5 × 10^5^ CT26 tumor cells subcutaneously. 10 days later, following establishment of tumors, mice were administered 50 µg of Alb-IL2. 1 day later, mice were treated with 5 × 10^6^ *Salmonella* intravenously. Mice were treated again with 50 µg of Alb-IL2 on day 17. Two weeks after the initiation of the treatment, tumor infiltrating lymphocytes were isolated and T cells quantified with flow cytometry. The quantification of (**a**) T cells, (**b**) CD4 T cells, and (**c**) CD8 T cells for the indicated treatment groups. N = 5 mice per group. Data is represented by mean ± SEM. P values were calculated by ordinary one-way ANOVA with the Tukey–Kramer multiple comparison test, and P < 0.05 is considered statistically significant. *,**,***, and **** indicate p values less than 0.05, 0.01, 0.001, and 0.0001, respectively

Next, we aimed to explore the effects that the combination treatment has on T cell responses in vivo. The induction of optimal antitumor immunity relies on both CD4 and CD8 T cells. CD4 T cells are crucial for the priming and activation of the effector CD8 T cells [19], and CD8 + T cells are critical to adaptive immune responses because they recognize and respond to intracellular pathogens and malignant cells by virtue of peptide presentation on MHC class I molecules. For that reason, the ability to generate a potent cytotoxic CD8 + T cell response is critical for the efficacy of cancer immunotherapeutic strategies. The presence of T cells in the tumor, specifically cytotoxic CD8 T cells, has been associated with improved clinical outcomes [20]. To further understand the observed anti-tumor effects elicited by *Salmonella* + Alb-IL2 treatment, we characterized the resulting T cells in CT26 tumor-bearing mice treated with single agent or combination therapy. We analyzed lymphocytes isolated from the tumor tissue with flow cytometry. Analysis revealed significant T cell infiltration, measured as total CD3 + T cells, following *Salmonella* + Alb-IL2 treatment (Fig. 4a). In comparison, mice treated with *Salmonella* or Alb-IL2 alone had minimal T cell present in the TME. Moreover, frequencies of both CD4 + and CD8 + T cells were elevated in the *Salmonella* + Alb-IL2 group (Fig. 4b,c). This supports that treatment with *Salmonella* + Alb-IL2 drives the proliferation of CD4 and CD8 T cell populations which likely contributes to the observed anti-cancer effects.

### *Salmonella* + Alb-IL2 treatments enhances the effector function of T lymphocytes

**Fig. 5:**
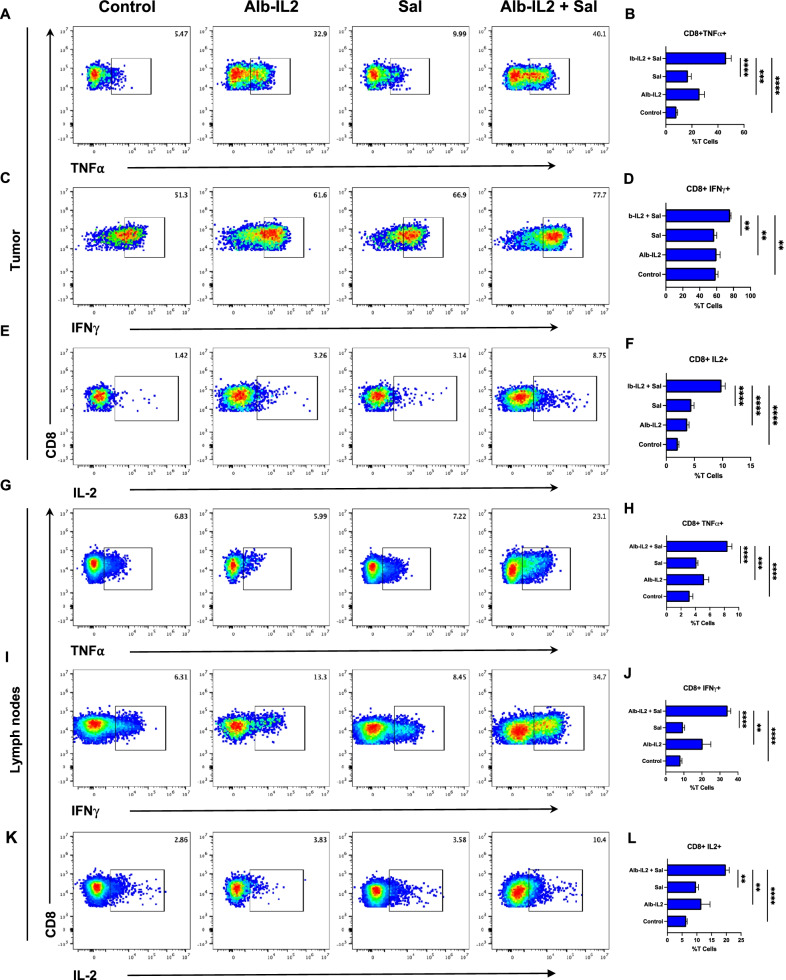
*Salmonella* + Alb-IL2 treatment enhance the effector function of T cells. BALB/c mice were inoculated with 5 × 10^5^ CT26 tumor cells subcutaneously. 10 days later, mice were administered 50ug of Alb-IL2 intravenously. 1 day later, mice were treated with 5 × 10^6^ *Salmonella* intravenously. Mice were treated again with 50ug of Alb-IL2 on day 17. Two weeks after the initiation of the treatment, tumor-infiltrating CD8 T cells were isolated and assessed for pro-inflammatory cytokine production. Representative flow gating and quantification of (**a**, **b**) CD8 + TNFα + , (**c**, **d**) CD8 + IFNγ + , and (**e**, **f**) CD8 + IL2 + for the indicated treatment groups. Similarly, CD8 T cells from the tumor draining lymph nodes were isolated and assessed. Representative flow gating and quantification of (**g**, **h**) CD8 + TNFα + , (**i**, **j**) CD8 + IFNγ + , and (**k**, **l**) CD8 + IL2 + in the tumor draining lymph nodes for the indicated treatment groups. N = 5 mice per group. Data is represented by mean ± SEM. P values were calculated by ordinary one-way ANOVA with the Tukey–Kramer multiple comparison test, and P < 0.05 is considered statistically significant. *,**,***, and **** indicate p values less than 0.05, 0.01, 0.001, and 0.0001, respectively

To better understand the mechanisms that contributed to the superior tumor control of the combination treatment, lymphocytes were isolated from tumor and draining lymph nodes of CT26 tumor-bearing mice and used for immune cell analysis by flow cytometry. Specifically, to address functional capabilities of the infiltrating lymphocytes, proinflammatory cytokine production was analyzed in CD8 T cells (Additional file 1: Fig. S4). Pro-inflammatory cytokine production including tumor necrosis factor α (TNFα) (Fig. 5a, b), IFNγ (Fig. 5c, d), and IL-2 (Fig. 5e,f) were significantly elevated in tumor-infiltrating CD8 + T cells from *Salmonella* + Alb-IL2 treated mice compared to mice treated with *Salmonella* or Alb-IL2 alone. A similar phenomenon was also observed in the draining lymph nodes (Fig. 5g-l). Furthermore, we observed a decrease in Tregs in the *Salmonella* + Alb-IL2 treatment group compared to the controls (Additional file 1: Fig. S5). These results suggest that *Salmonella* + Alb-IL2 combination therapy drives T cell immunity, specifically expanding the effector function of T cells while decreasing their regulatory capabilities, leading to the improved tumor control.

### T cell depleting antibodies abrogate therapeutic efficacy of *Salmonella* + Alb-IL2 combination therapy

**Fig. 6:**
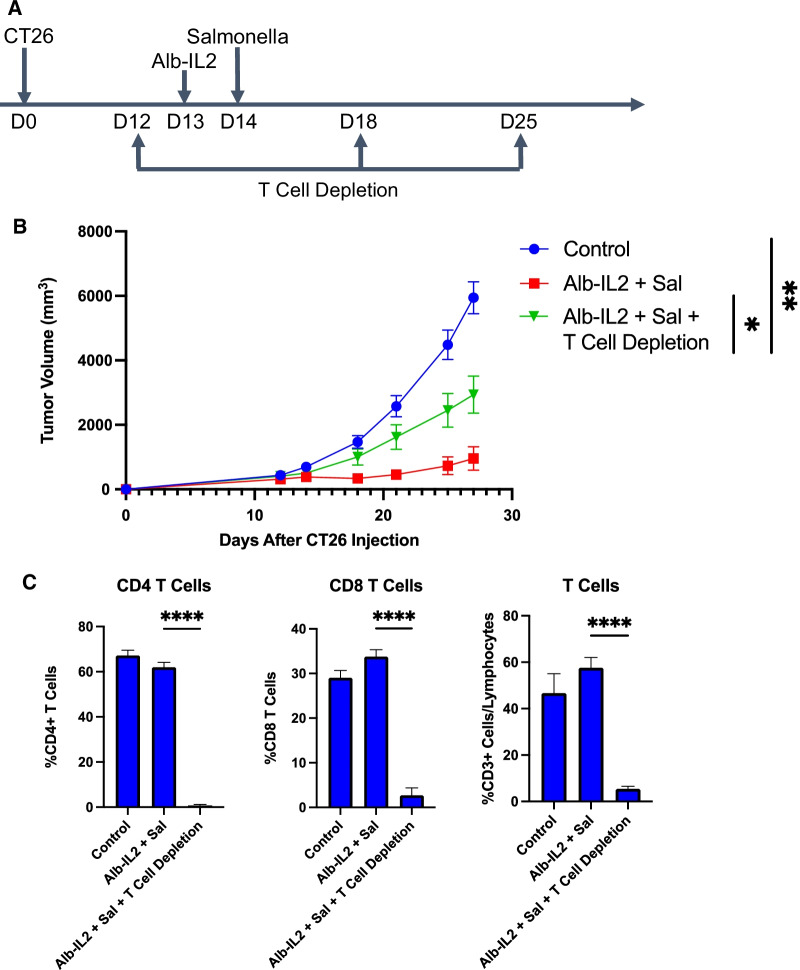
T cell-dependence of *Salmonella* + Alb-IL2 combination therapy. (**a**) Schematic of experimental design. Briefly, BALB/c mice were inoculated with 5 × 10^5^ CT26 tumor cells subcutaneously. On day 12, 18, and 25 mice were treated with 100 µg of anti-mouse CD4 and CD8 antibodies, each. 50ug of Alb-IL2 was treated on day 13 and 5 × 10^6^ *Salmonella* SL7207 the following day. (**b**) Tumor growth following the described treatment protocol for the indicated treatment groups. PBMCs were collected on day 27 and were assessed for T cell frequency using flow cytometry. (**c**) Frequency of the indicated cell populations

Since we observed that *Salmonella* + Alb-IL2 treated mice had an increase in the frequency of T cells and enhanced production of pro-inflammatory cytokines by CD8 T cells, we sought to characterize how the alteration of these factors impacts the therapeutic efficacy of *Salmonella* + Alb-IL2 treatment. CT26 tumor-bearing BALB/c mice received three doses of T cell depleting antibodies while being administered Alb-IL2 and *Salmonella*, as indicated in the schematic (Fig. 6a). To verify that T cells have been depleted from the mice, PBMCs were collected from antibody-treated mice on day 28 and analyzed using flow cytometry (Additional file 1: Fig. S6). Minimal T cells were observed in the depletion groups, signifying their successful depletion (Fig. 6c). We administered *Salmonella* + Alb-IL2 to both T-cell-depleted mice and normal tumor-bearing mice and observed a dramatic reduction in tumor control in T-cell-depleted mice, whereas control mice demonstrated robust anticancer effects (Fig. 6b). This reaffirms the dependence of the demonstrated tumor control on T cell immunity.

## Discussion

Advances with chemotherapy, radiation and surgical resection have had profound impacts on cancer treatment and for some cancers, is no longer a death sentence as a result. Despite improved disease outcomes, the ability to localize drugs to the TME, generate a potent immune response that is primed against tumor tissue, and limit adverse effects such as off-target activity remain challenges to generating a broadly applicable cancer treatment. The development of engineered strains of bacteria have alleviated the initial issues of toxicity and localization and have made bacteria more suitable for therapeutic development to address some of the shortcomings with current therapeutic modalities.

Engineered strains of bacteria, such as *Salmonella* SL7207, readily colonize and proliferate in hypoxic tissue such as tumors [5]. Consistent with recent findings, our IVIS data demonstrated that after systemic administration, *Salmonella* SL7207 exclusively localized in tumors and was undetectable in other tissues. This property is advantageous to a therapeutic strategy because bacteria contain multiple pathogen associated molecular patterns that are capable of activating host pattern recognition receptors (PRR’s). As a consequence of PRR activation, dendritic cells undergo maturation and upregulate MHC molecules on their surface to facilitate presentation of antigen to the T cells, which are an important component of adaptive immune responses [21]. Induction of immunity specifically in the tumor due to preferential bacterial colonization positions intratumoral dendritic cells for effective T cell priming against tumor antigens.

Interestingly, our data demonstrated a synergistic effect with *Salmonella* and Alb-IL2 immunotherapy that resulted in robust tumor control that was dependent on T cell immunity. When tumor bearing mice were treated with *Salmonella* + Alb-IL2, we observed increased frequencies of intra-tumoral CD4 and CD8 T cells as well as elevated levels of pro-inflammatory cytokines (IFNγ, TNFα, IL-2) in the tumor associated CD8 T cells. Control mice or mice treated with either monotherapy failed to expand T cells and mice succumbed to tumor burden. We reasoned that bacteria, which are inherently immunogenic, were able to induce local inflammation that was further augmented by Alb-IL2. Alb-IL2 has been shown to preferentially accumulate in secondary lymphoid tissue and tumors given its slower rate of plasma clearance, which increases the likelihood of IL-2 to encounter and bind to IL-2R + lymphocytes [14]. Although *Salmonella* and Alb-IL2 monotherapies resulted in partial tumor control, we think that the lack of T cells entering the TME was a contributing factor to the eventual tumor outgrowth. In line with this, the dependence on T cell immunity was evidenced by the loss of tumor controlling abilities when CD4 and CD8 T cells were depleted prior to administration of *Salmonella* + Alb-IL2. We performed this experiment as a proof of principal to show the importance of T cells in the observed antitumor effects. In future studies, we plan to explore the role of CD4 and CD8 T cells separately and explore their respective roles in the antitumor immunity mediated by *Salmonella* + Alb-IL2. Furthermore, before additional preclinical studies, a kinetic study would be appropriate to determine the localization of *Salmonella* at various time points. For the future, we aim to assess the luciferase signals of the *Salmonella* over time to better inform their clinical dosing and usage.

It has been established that the presence of immune suppressing cells, such as CD4 + FoxP3 + T regulatory cells (Tregs), in the TME can impede T cell immunity and contribute to tumor progression [22]. Tregs express high levels of CD25 and are dependent on IL-2 for their survival and function in the periphery [23]. Alb-IL2 may expand this unintended cell population as consequence of this treatment strategy. Incorporating an IL-2 biased agonist in our Alb-IL2 system could circumvent this issue if it were to arise. IL-2 biased agonists contain mutations eliminating CD25 binding and enhancing binding to the CD122/CD132 subunits of the IL-2 receptor. Existing literature has showed that IL-2 biased agonists preferentially expand CD8 T cells and NK cells over Tregs and contribute to improved anti-tumor responses compared to IL-2 [24–26]. This could serve as a direction for future studies exploring *Salmonella* + Alb-IL2 therapeutically.

## Conclusions

The development of broadly applicable, off-the-shelf cancer immunotherapeutic modalities is highly sought after. Our work describes the use of *Salmonella* and Alb-IL2 as a novel approach to drive local inflammation in the TME and potent anti-tumor T cell responses. Our results clearly demonstrated the ability of *Salmonella* + Alb-IL2 to control tumor growth and provide durable immunity. In summary, *Salmonella* + Alb-IL2 has potential utility as an off-the-shelf therapy for treating cancer.

## Supplementary Information

**Additional file 1: Figure S1.** Spider plots for the indicated treatment groups. BALB/c mice were inoculated with 5e5 CT26 tumor cells subcutaneously. 10 days later, following establishment of tumors, mice were administered 50ug of Alb-IL2. 1 day later, mice were treated with 5e6 Salmonella intravenously. Mice were treated again with 50ug of Alb-IL2 on day 17. **Figure S2.** Growth, survival, and body weight curve of TC-1 tumor-bearing mice treated with Salmonella + Alb-IL2. C57BL/6 mice were inoculated with 5e5 TC-1 tumor cells subcutaneously. 10 days later, following establishment of tumors, mice were administered 50ug of Alb-IL2. 1 day later, mice were treated with 5e6 Salmonella intravenously. Mice were treated again with 50ug of Alb-IL2 on day 17. **Figure S3.** Bioactivity of Alb-IL2. Memory CD8+ T Cells generated in house were rested, then stimulated with our Alb-IL2 alongside relevant controls as indicated in the figure. 20 minutes later, the cells were fixed in methanol, collected, and analyzed for pSTAT5 levels by phosphoflow. Stimulation condition is indicated on the right. Level normalized to mode is shown on the Y axis. **Figure S4.** Flow gating strategy for identification of T cell populations and pro-inflammatory cytokine production. Lymphocytes from the tumor and draining lymph nodes were isolated two weeks after the initiation of treatment and analyzed using flow cytometry. This gating strategy allowed for the identification of T cell populations and observation of their effector functions. **Figure S5.** Quantification of additional immune cell populations in Salmonella + Alb-IL2 treated mice. Lymphocytes from the blood were isolated two weeks after the initiation of treatment and analyzed using flow cytometry. Quantification of a) CD8+ CD44+ b) CD8+ Ki67+ c) CD8+ CD122+ d) CD4+ FoxP3+ and e) NK1.1+ NK Cells. **Figure S6.** Representative flow gating of T cell depletion. 27 days post CT26 tumor inoculation in BALB/c mice,  3 doses of T cell depleting antibodies were administered. PBMCs were collected and stained for flow cytometric analysis. CD4 and CD8 T cell populations and gating strategy is shown. 

## Data Availability

Available from the corresponding authors upon request.

## Abbreviations

IL-2: Interleukin-2
Alb-IL2: Albumin-IL-2
FcRN: Neonatal Fc receptor
TME: Tumor microenvironment
PRR: Pattern recognition receptor
Treg: T regulatory cell
IVIS: In vivo imaging system
TNFα: Tumor necrosis factor α
INFγ: Interferon γ
LN: Lymph node
MHC: Major histocompatibility complex
FACS: Fluorescence-activated cell sorting
