# Supplementary material for: Localization of Salmonella and albumin-IL-2 to the tumor microenvironment augments anticancer T cell immunity
Source: J Biomed Sci. 2022 Aug 12;29:57. doi: 10.1186/s12929-022-00841-y (PMC9373295; doi:10.1186/s12929-022-00841-y)
Supplement: Supplementary file 1 — Additional file 1: Figure S1. Spider plots for the indicated treatment groups. BALB/c mice were inoculated with 5e5 CT26 tumor cells subcutaneously. 10 days later, following establishment of tumors, mice were administered 50ug of Alb-IL2. 1 day later, mice were treated with 5e6 Salmonella intravenously. Mice were treated again with 50ug of Alb-IL2 on day 17. Figure S2. Growth, survival, and body weight curve of TC-1 tumor-bearing mice treated with Salmonella + Alb-IL2. C57BL/6 mice were inoculated with 5e5 TC-1 tumor cells subcutaneously. 10 days later, following establishment of tumors, mice were administered 50ug of Alb-IL2. 1 day later, mice were treated with 5e6 Salmonella intravenously. Mice were treated again with 50ug of Alb-IL2 on day 17. Figure S3. Bioactivity of Alb-IL2. Memory CD8+ T Cells generated in house were rested, then stimulated with our Alb-IL2 alongside relevant controls as indicated in the figure. 20 minutes later, the cells were fixed in methanol, collected, and analyzed for pSTAT5 levels by phosphoflow. Stimulation condition is indicated on the right. Level normalized to mode is shown on the Y axis. Figure S4. Flow gating strategy for identification of T cell populations and pro-inflammatory cytokine production. Lymphocytes from the tumor and draining lymph nodes were isolated two weeks after the initiation of treatment and analyzed using flow cytometry. This gating strategy allowed for the identification of T cell populations and observation of their effector functions. Figure S5. Quantification of additional immune cell populations in Salmonella + Alb-IL2 treated mice. Lymphocytes from the blood were isolated two weeks after the initiation of treatment and analyzed using flow cytometry. Quantification of a) CD8+ CD44+ b) CD8+ Ki67+ c) CD8+ CD122+ d) CD4+ FoxP3+ and e) NK1.1+ NK Cells. Figure S6. Representative flow gating of T cell depletion. 27 days post CT26 tumor inoculation in BALB/c mice, 3 doses of T cell depleting antibodies were adm [file 12929_2022_841_MOESM1_ESM.pptx]

## Slide 1
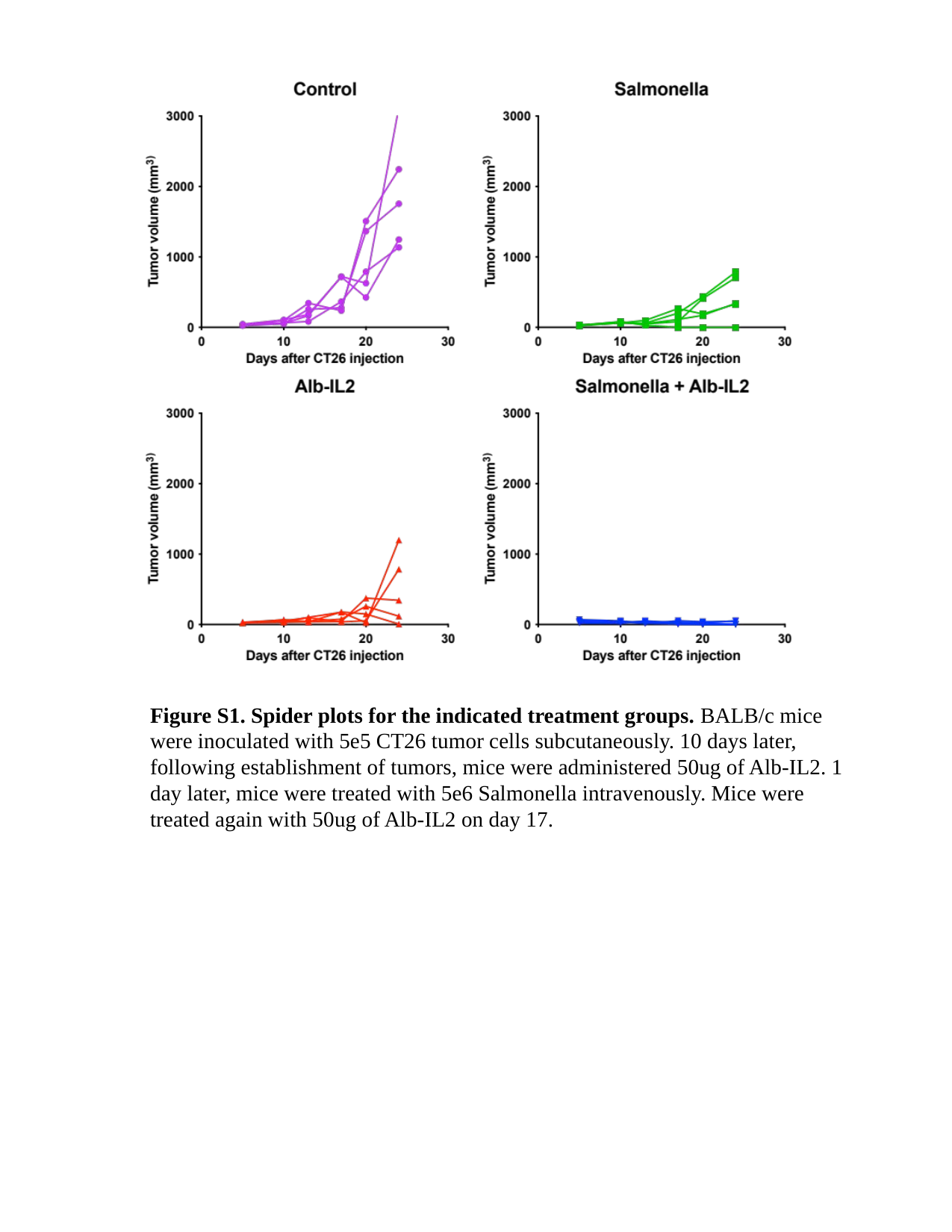

Figure S1. Spider plots for the indicated treatment groups. BALB/c mice were inoculated with 5e5 CT26 tumor cells subcutaneously. 10 days later, following establishment of tumors, mice were administered 50ug of Alb-IL2. 1 day later, mice were treated with 5e6 Salmonella intravenously. Mice were treated again with 50ug of Alb-IL2 on day 17.

## Slide 2
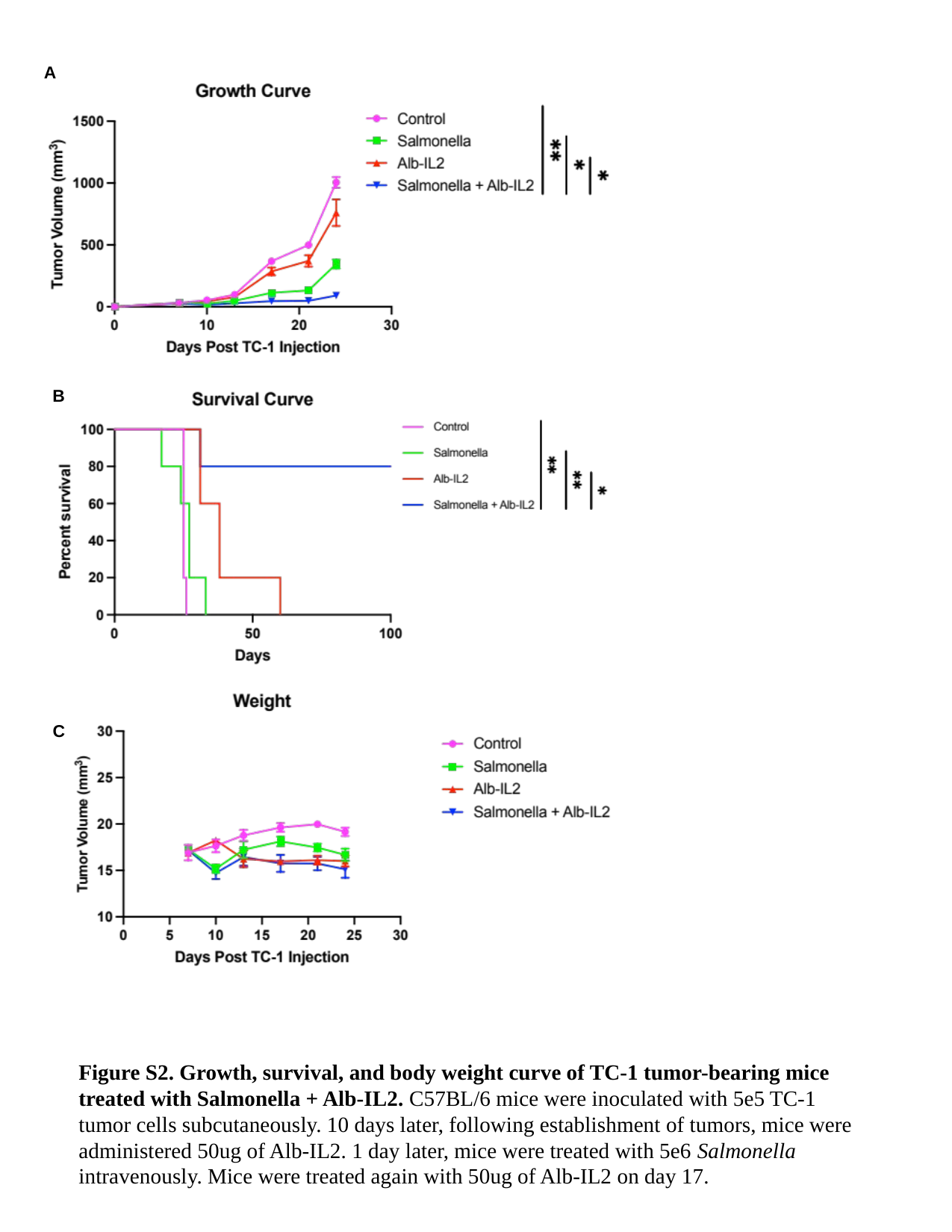

A
B
C
Figure S2. Growth, survival, and body weight curve of TC-1 tumor-bearing mice treated with Salmonella + Alb-IL2. C57BL/6 mice were inoculated with 5e5 TC-1 tumor cells subcutaneously. 10 days later, following establishment of tumors, mice were administered 50ug of Alb-IL2. 1 day later, mice were treated with 5e6 Salmonella intravenously. Mice were treated again with 50ug of Alb-IL2 on day 17.

## Slide 3
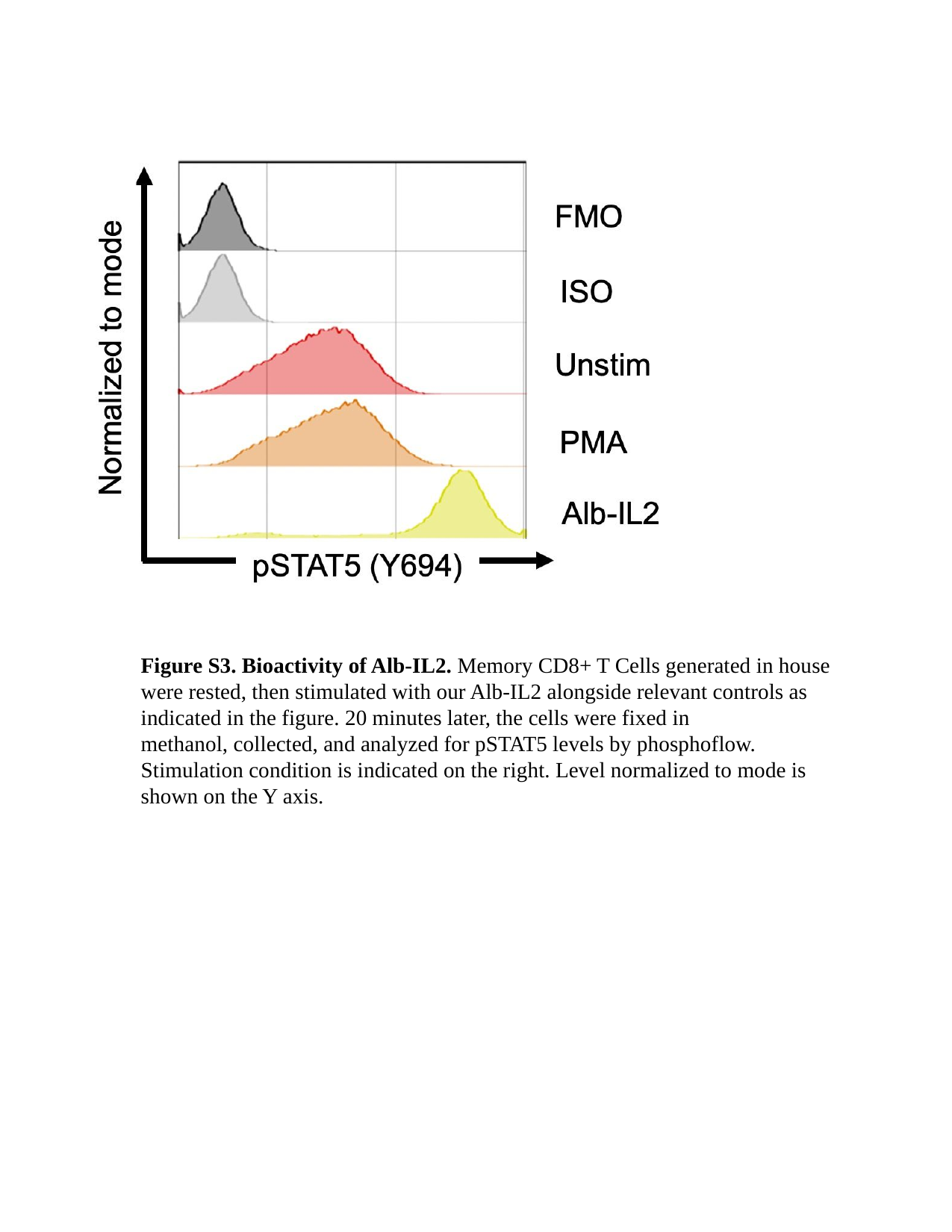

Figure S3. Bioactivity of Alb-IL2. Memory CD8+ T Cells generated in house were rested, then stimulated with our Alb-IL2 alongside relevant controls as indicated in the figure. 20 minutes later, the cells were fixed in methanol, collected, and analyzed for pSTAT5 levels by phosphoflow. Stimulation condition is indicated on the right. Level normalized to mode is shown on the Y axis.

## Slide 4
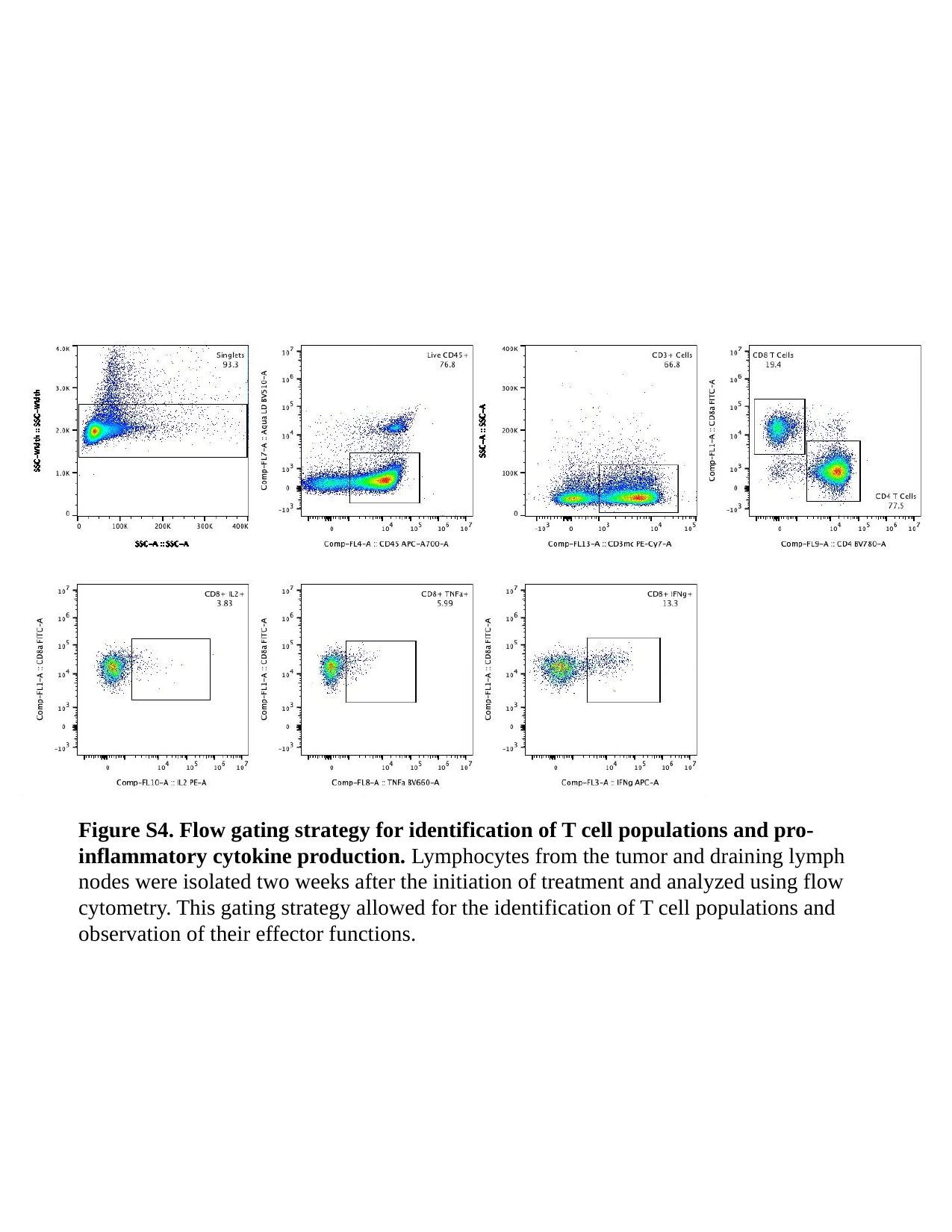

Figure S4. Flow gating strategy for identification of T cell populations and pro-inflammatory cytokine production. Lymphocytes from the tumor and draining lymph nodes were isolated two weeks after the initiation of treatment and analyzed using flow cytometry. This gating strategy allowed for the identification of T cell populations and observation of their effector functions.

## Slide 5
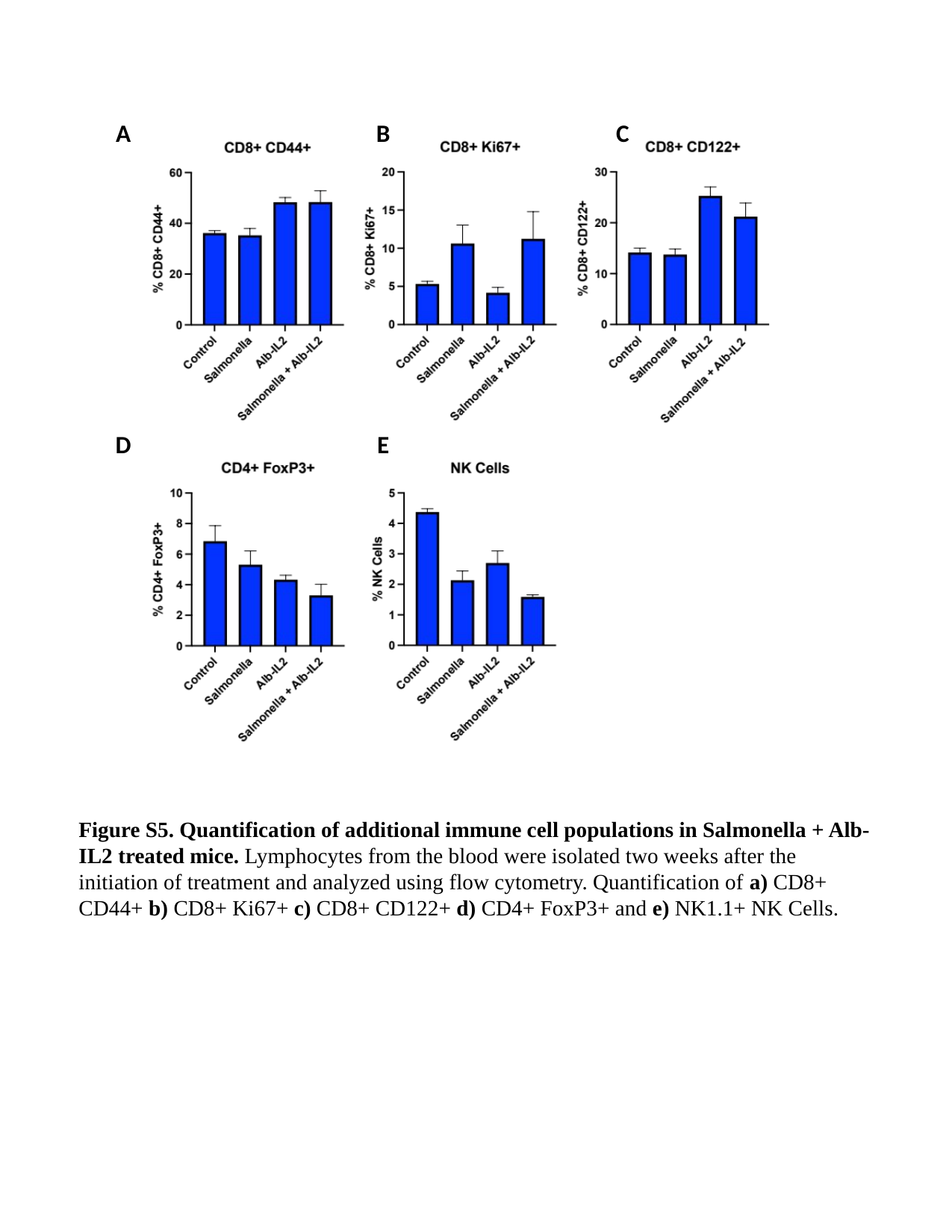

A
B
C
D
E
Figure S5. Quantification of additional immune cell populations in Salmonella + Alb-IL2 treated mice. Lymphocytes from the blood were isolated two weeks after the initiation of treatment and analyzed using flow cytometry. Quantification of a) CD8+ CD44+ b) CD8+ Ki67+ c) CD8+ CD122+ d) CD4+ FoxP3+ and e) NK1.1+ NK Cells.

## Slide 6
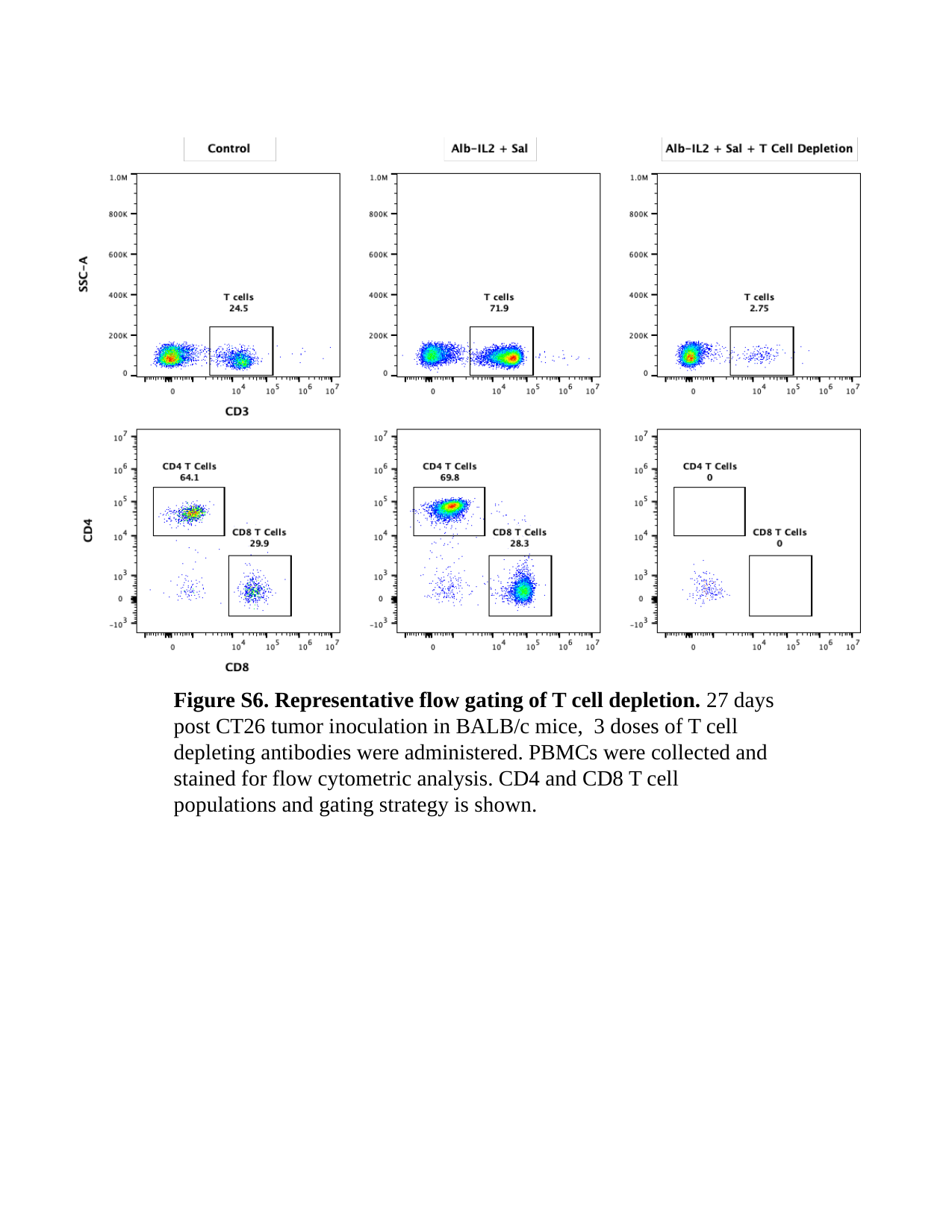

Figure S6. Representative flow gating of T cell depletion. 27 days post CT26 tumor inoculation in BALB/c mice,  3 doses of T cell depleting antibodies were administered. PBMCs were collected and stained for flow cytometric analysis. CD4 and CD8 T cell populations and gating strategy is shown.
